# Correlation of muscle strength, working memory, and activities of daily living in older adults

**DOI:** 10.3389/fnagi.2024.1453527

**Published:** 2024-09-20

**Authors:** Jinlin Liao, Jing Wang, Shuqi Jia, Zhidong Cai, Hairong Liu

**Affiliations:** ^1^College of Physical Education and Health, Longyan University, Longyan, China; ^2^School of Sports and Health of Shanghai Lixin University of Accounting and Finance, Shanghai, China; ^3^Shanghai University of Sport, Shanghai, China; ^4^Sports Department of Suzhou University of Science and Technology, Suzhou, China; ^5^Physical Education Department of Shanghai International Studies University, Shanghai, China

**Keywords:** muscle strength, working memory, activities of daily living, older adults, mediation model

## Abstract

**Objective:**

This study aims to investigate the relationship between muscle strength, working memory, and activities of daily living (ADL) in older adults. Additionally, it seeks to clarify the pathways and effects of working memory in mediating the relationship between muscle strength and ADL.

**Methods:**

Using a cross-sectional study design, we recruited 245 older adults individuals from nursing homes. We collected data on grip strength, the 30-s sit-to-stand test, the N-back task, and ADL. The data were analyzed using independent sample t-tests, χ2 tests, correlation analysis, and structural equation modeling.

**Results:**

Grip strength significantly influenced ADL (effect size = −0.175, 95% CI: −0.226 to −0.124). Grip strength also had a significant direct effect on ADL (effect size = −0.114, 95% CI: −0.161 to −0.067). The 1-back task correct rate significantly mediated the relationship between grip strength and ADL (effect size = 0.054, 95% CI: −0.084 to −0.029). The 30-s sit-to-stand test significantly impacted ADL (effect size = −0.280, 95% CI: −0.358 to −0.203). It also had a significant direct effect on ADL (effect size = −0.095, 95% CI: −0.183 to −0.007). The 1-back task correct rate significantly mediated the relationship between the 30-s sit-to-stand test and ADL (effect size = −0.166, 95% CI: −0.236 to −0.106).

**Conclusion:**

There exists a strong correlation between muscle strength, working memory, and ADL. Increased muscle strength leads to better ADL performance and improved working memory tasks. Low cognitive load working memory tasks can mediate the relationship between muscle strength and ADL. Regular physical exercise can enhance muscle strength, slow down the decline of working memory, thereby maintaining or improving ADL in older adults.

## 1 Introduction

Around 15% of the global population experiences a decline in their activities of daily living (ADL) (World Health Organization, 2011). In individuals aged 60 and older, this decline occurs at a rate of 46.1%, increasing with age (Wei et al., 2019). ADL includes basic physical actions required for daily independent living, such as eating, dressing, getting in and out of bed, using the toilet, and bathing (Katz et al., 1963; Shuqing et al., 2022). A decline in ADL is a significant contributor to disability, dependence, and mortality (Wang et al., 2020). By 2030, it is estimated that over 77 million older adults individuals in China will experience ADL decline, accounting for more than 57% of the total disabled population (Luo et al., 2020).

Working memory is closely linked to ADL and May serve as an important predictor of ADL. It is a limited-capacity system that temporarily stores and processes information during cognitive tasks, essential for reasoning, decision-making, and behavior (Baddeley, 2012; Cai Zhi-Dong et al., 2022). Studies have shown that a decline in ADL is strongly associated with impaired cognitive function, and executive function is a key predictor of ADL (Li et al., 2008; Mansbach and Mace, 2019). Friedman et al. (2006) found that, compared to other components of executive function like inhibition and switching, working memory almost completely modulates age-related changes in fluid intelligence, which is closely related to ADL (Chen and Li, 2007). Both fluid and crystallized intelligence are significant predictors of ADL (Jacob et al., 2020), indicating that working memory could be an important predictor of ADL.

Muscle strength, working memory, and ADL are closely interconnected. Aging leads to degenerative changes in bodily functions, initially manifested by a decline in muscle strength (Pinsker et al., 2011). Muscle strength peaks around age 30, declines by an average of 16% by age 40, and falls by 40.9% in those over 60, leading to decreased ADL (Keller and Engelhardt, 2013). Muscle strength is crucial for performing ADL, and its decline can lead to ADL impairment; low muscle strength is an important predictor of ADL disability (Janssen et al., 2002; Hairi et al., 2010; Zicai et al., 2017). Therefore, enhancing muscle strength can prevent ADL decline. Grip strength and the 30-s sit-to-stand test are widely used to assess muscle strength. Grip strength measures upper limb muscle strength and reflects overall muscle strength and physical function (Rantanen et al., 1999); the 30-s sit-to-stand test measures lower limb muscle strength and reflects balance, coordination, and endurance (Jones et al., 1999; Sijie et al., 2012). From a cognitive perspective, age-related brain deterioration and functional impairment lead to cognitive decline, with working memory considered the core of cognitive activities (Baddeley, 1992; Elliott et al., 2011). Working memory performance peaks around age 30 and significantly declines after age 60 (Wang et al., 2011). It is crucial for maintaining personal functionality and independent living. The decline in muscle strength and working memory occurs synchronously. Cohort studies show that grip strength can predict brain health (total brain volume, white matter volume) and cognitive function in older adults (Dercon et al., 2021). Chen et al. found that greater quadriceps strength is associated with better cognitive performance in older adults (Chen et al., 2015). Muscle strength positively correlates with working memory (Firth et al., 2018a). Higher grip strength is associated with better overall cognitive function, including declarative memory, working memory, and attention (Filardi et al., 2022), and lower limb muscle strength is closely related to attention and working memory (Scherder et al., 2010).

Previous research extensively covers the correlation between muscle strength, working memory, and ADL. Muscle strength can predict working memory, and working memory is a significant predictor of ADL. Given the close relationship among the three, does working memory mediate the relationship between muscle strength and ADL? If so, how does working memory regulate muscle strength to affect ADL? Do the effects of upper and lower limb muscle strengths differ, and are their pathways consistent? Addressing these questions is crucial. This study uses a cross-sectional design to explore the relationships among muscle strength, working memory, and ADL in older adults. It aims to clarify how working memory mediates the effect of muscle strength on ADL and to provide evidence for maintaining or improving ADL in older adults.

## 2 Research methods

### 2.1 Research subjects

Participants were older adults individuals aged 60 and above. Sample size estimation was based on the Monte Carlo power analysis principle for mediating effects, using an online tool, “Monte Carlo Power Analysis for Indirect Effects,” developed by Alexander M. Schoemann (Contact), Aaron J. Boulton, and Stephen D. Short (URL: https://schoemanna.shinyapps.io/mc_power_med/). Effect size was set based on previous literature (Jefferson et al., 2006; Irisawa and Mizushima, 2022; Ferguson, 2016), with a statistical power of 0.8 achieved for mediating effects with a sample size of 196. Considering a 10% sample loss rate, 216 participants were targeted.

**Figure 1:**
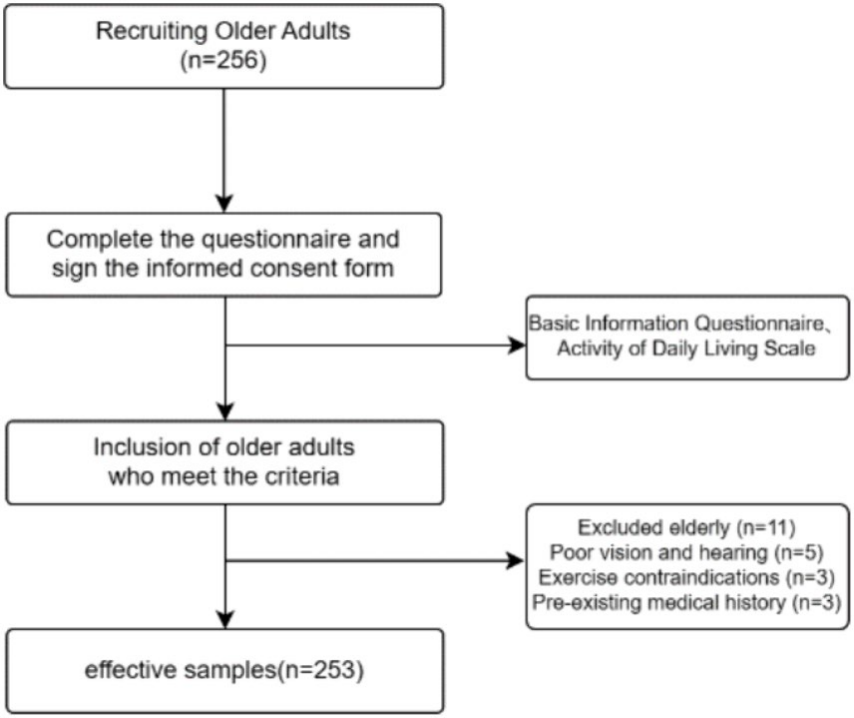
Flow chart of subject recruitment.

Convenience sampling was used to randomly select older adults individuals aged 60 and above from a nursing home in Shanghai. Inclusion criteria included right-handedness, normal verbal communication, the ability to see and hear, sufficient mental status to participate, no mild cognitive impairment or dementia, no use of anti-cognitive impairment medication, no major organic diseases, no exercise contraindications, and no vigorous exercise or consumption of caffeinated or alcoholic beverages within 24 h before testing. Participants filled out a basic information questionnaire and an ADL scale. The recruitment process is shown in Figure 1. All participants volunteered for the study and signed informed consent forms. This study complies with the latest version of the Helsinki Declaration’s ethical requirements and has been approved by the Ethics Committee of Shanghai University of Sport (102772020RT060).

### 2.2 Test procedure

**Figure 2:**
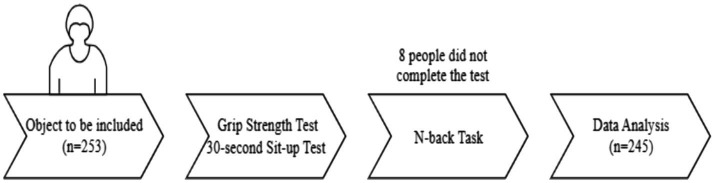
Flow chart of the test procedure.

The test was conducted during the period of 13:30–16:30. After explaining the test procedure to the subjects, a grip strength test, a 30-s sit-up test, and a working memory task test were performed. The test procedure is presented in Figure 2.

### 2.3 Test tools

#### 2.3.1 Basic information questionnaire

The basic information questionnaire includes name, age, gender, height, weight, preferred hand, any contraindications to exercise, past medical history, and medication use.

#### 2.3.2 Activity of daily living scale (ADL)

Developed by Lawton and Brody in 1969, this scale is used to assess participants’ ADL. It consists of 14 items covering two dimensions: physical ADL and instrumental ADL. The scoring system ranges from 1 to 4 points, with 1 point indicating no impairment. A total score of 14 signifies completely normal ADL, while scores above 14 indicate a decline in ADL, with higher scores representing greater impairment. The highest possible score is 56. The ADL scale used in this study has high internal consistency, with a Cronbach’s alpha coefficient of 0.93 (Jian Wen-Jia et al., 2014).

#### 2.3.3 Grip strength test

Grip strength reflects overall muscle strength and physical function and is highly practical and sensitive (Ma Xin, 2021). Participants stood with their elbow extended and arm resting at their side, gripping the dynamometer. They exerted maximal force for 3 to 5 s. Both hands were tested three times each, with a 30-s interval between tests. The highest value was recorded as the grip strength.

#### 2.3.4 30-s sit-up test

30-s sit-up can indicate the muscle strength of the lower limbs of the older adults, which has good reliability and validity (Bo Wen-Xi et al., 2020). The subjects sat in a chair with arms in a crossed position in front of the chest. The test began with the body standing as upright as possible. The subjects then sat down until the back touched the back of the chair once, then stood up and repeated the action for 30 s. Test 3 times and take the average.

#### 2.3.5 N-back task

In this study, the N-back task paradigm was adopted to test working memory, which was programmed using the psychological experiment software E-prime 2.0, where the stimulus type was numeric and the subjects performed the stimulus keystroke response on the computer as required, and the response time and correctness of the N-back task were recorded. The N-back of this experiment was designed with two different loadings of cognitive tasks, including 1-back and 2-back. The 1-back task asked the subjects to determine if the current number was the same as the previous number, starting with the second number, and press “1” for the same and “2” for different. The 2-back task required the subject to judge whether the current number was the same as the second number before it, starting with the third number, and press “1” for the same and “2” for different. The instruction and exercise were set before the start of the formal experiment, and each task was repeated 5 times. At the beginning of the experiment, a “+” was presented for 500 ms for the subject to maintain attention, then 10 random numbers between 0 and 9 were shown for 500 ms, and finally a blank screen appeared for 2000 ms. If no response was made during the period of the blank screen, the next numerical stimulus was automatically presented, and the prompt “30s break” appeared at the end of each task.

### 2.4 Statistical analysis

Data were statistically analyzed using SPSS 26.0. ADL scores of 14 indicated normal ADL, while scores above 14 indicated impaired ADL. Descriptive statistics for continuous variables were presented as mean ± standard deviation, with results rounded to three decimal places. Independent sample t-tests were used for group comparisons, and χ^2^ tests were used for categorical data comparisons described as *n* (%). Pearson correlation analysis was conducted to explore the relationship between muscle strength, working memory, and ADL. The mediation effect of working memory between muscle strength and ADL was analyzed using Model 4 (parallel mediation model) of PROCESS (Hayes, 2013), with age and years of education as control variables. Path analysis parameters were estimated using the Bootstrap method with 5,000 samples and a 95% confidence interval. A confidence interval not containing zero was considered statistically significant. All statistical inferences were performed using two-tailed tests, with an *α* level of 0.05 indicating statistical significance.

## 3 Results

### 3.1 Differences in muscle strength and working memory in older adults with different ADL scores

As shown in Table 1, a total of 245 older adults participated in the study, with an average age of 76.22 ± 7.83 years. ADL scores of 14 indicated normal ADL, while scores above 14 indicated impaired ADL. Compared to those with normal ADL, Older adults individuals with impaired ADL showed statistically significant differences in age, years of education, grip strength, 30-s sit-to-stand, 1-back correct rate, 1-back response time, and 2-back correct rate (all *p* < 0.001). No significant differences were found in other variables (all *p* > 0.05).

**Table 1:** Comparison of differences in muscle strength and working memory in older adults with different ADL scores.

Variables	Scores of activities of daily living			Test of variability
	Whole (*n* = 245)	Normal activities of daily living (*n* = 118)	Reduced activities of daily living (*n* = 127)	
Age (year)	76.22 ± 7.83	73.63 ± 7.33	78.64 ± 7.52	*t* = −5.275, *P*<0.001
Height (m)	1.62 ± 0.08	1.63 ± 0.08	1.62 ± 0.07	*t* = 1.732, *p* = 0.085
Weight (kg)	61.36 ± 9.08	61.75 ± 9.38	61.00 ± 8.82	*t* = 0.642, *p* = 0.522
BMI (kg/m^2^)	23.22 ± 2.64	23.10 ± 2.57	23.34 ± 2.70	*t* = −0.695, *p* = 0.488
Years of education	8.14 ± 4.60	8.90 ± 4.26	7.44 ± 4.81	*t* = 2.504, *p* = 0.013
Gender				*χ^2^ = 0.019*, *p = 0.890*
Male	111	54	57	
Female	134	64	70	
Grip Strength (kg)	25.39 ± 8.44	29.04 ± 8.39	22.01 ± 6.97	*t* = 7.111, *P*<0.001
30-s sit-up (second)	14.91 ± 5.35	17.87 ± 5.41	12.16 ± 3.53	*t* = 9.704, *P*<0.001
1-back correct rate	0.75 ± 0.17	0.87 ± 0.10	0.64 ± 0.15	*t* = 14.056, *P*<0.001
1-back response time	905.90 ± 227.76	844.37 ± 211.19	963.07 ± 228.48	*t* = −4.213, *P*<0.001
2-back correct rate	0.61 ± 0.18	0.66 ± 0.17	0.56 ± 0.17	*t* = 4.934, *P*<0.001
2-back response time	1063.98 ± 232.01	1037.51 ± 228.99	1088.57 ± 232.98	*t* = −1.728, *P* = 0.085

### 3.2 The relationship between muscle strength, working memory, and ADL scores in older adults

**Figure 3:**
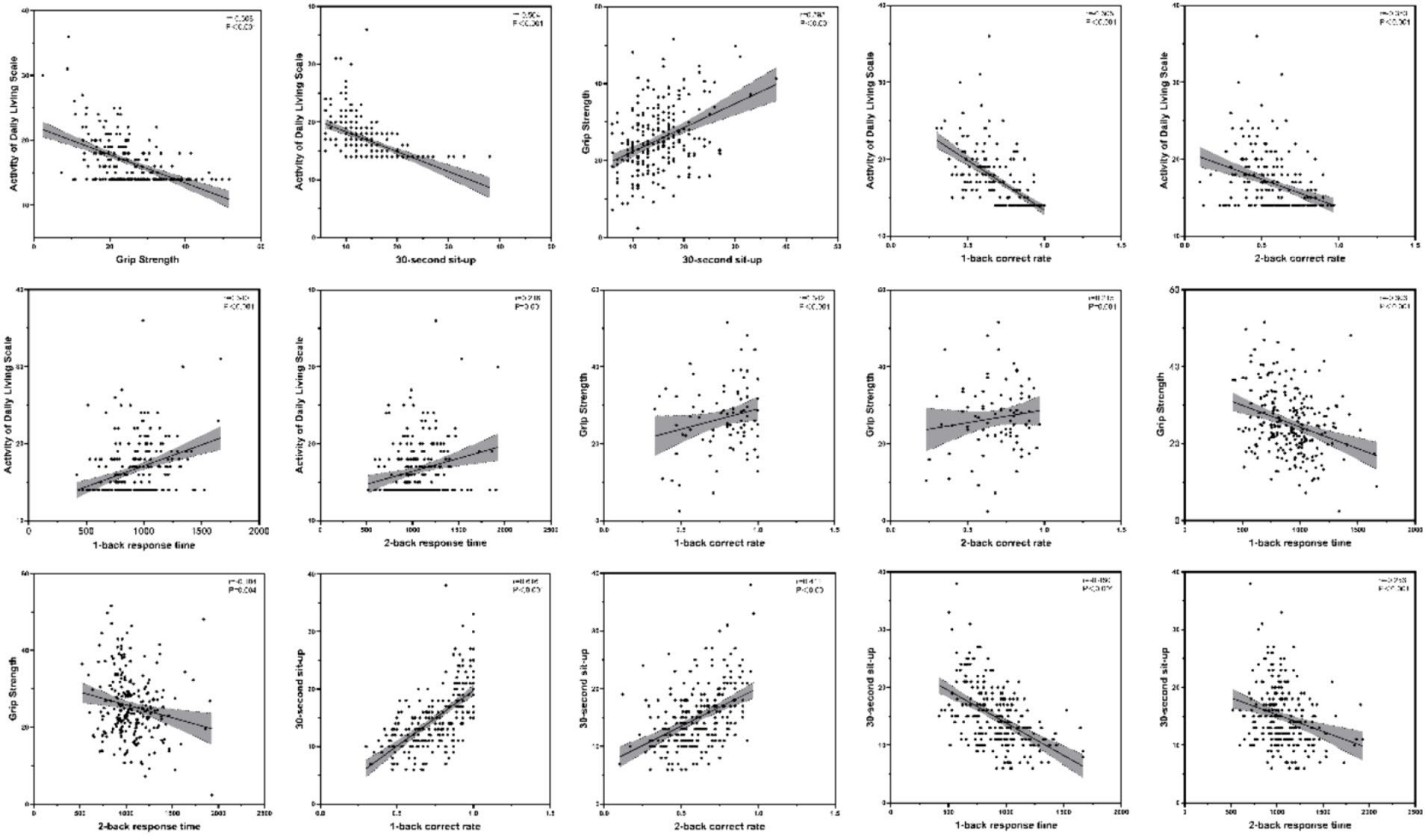
Correlations among variables (*N* = 245).

The Pearson correlation coefficient was used to examine the relationship between grip strength, 30-s sit-up performance, working memory, and ADL scores. The results showed significant negative correlations between grip strength and ADL scores (*r* = −0.508), 1-back response time (*r* = −0.303), and 2-back response time (*r* = −0.184) (all *p* < 0.01), while significant positive correlations were observed between grip strength and 30-s sit-up performance (*r* = 0.393), 1-back correct rate (*r* = 0.342), and 2-back correct rate (*r* = 0.215) (all *p* < 0.001). Similarly, 30-s sit-up performance showed significant negative correlations with ADL scores (*r* = −0.504), 1-back response time (*r* = −0.480), and 2-back response time (*r* = −0.253) (all *p* < 0.001), while significant positive correlations were found with 1-back correct rate (*r* = 0.616) and 2-back correct rate (*r* = 0.441) (all *p* < 0.001). ADL scores exhibited significant negative correlations with 1-back correct rate (*r* = −0.605) and 2-back correct rate (*r* = −0.353) (all *p* < 0.001), and significant positive correlations with 1-back response time (*r* = 0.340) and 2-back response time (*r* = 0.216) (all *p* < 0.001). Refer to Figure 3 for details. The results indicate that greater muscle strength in older adults is associated with lower ADL scores, faster response times, and higher correct rates in working memory tasks.

### 3.3 Construction and validation of structural relationship model among muscle strength, working memory, and activities of daily living

#### 3.3.1 Structural relationship model of grip strength, working memory, and activities of daily living

Controlling for age and years of education, grip strength was taken as the independent variable, 1-back correct rate, 1-back response time, 2-back correct rate, and 2-back response time as the mediating variables, and activities of daily living as the dependent variable. As shown in Table 2, grip strength positively predicted 1-back correct rate (*B* = 0.261, *p* < 0.001) and 2-back correct rate (*B* = 0.212, *p* < 0.01), while negatively predicting 1-back response time (*B* = −0.315, *p* < 0.001) and 2-back response time (*B* = −0.171, *p* < 0.05). Grip strength (*B* = −0.265, *p* < 0.001) and 1-back correct rate (*B* = −0.484, *p* < 0.001) negatively predicted ADL.

**Table 2:** Regression analysis of relationships among variables (*N* = 245).

Variables	Model 1		Model 2		Model 3		Model 4		Model 5	
	Beta	*t*	Beta	*t*	Beta	*t*	Beta	*t*	Beta	*t*
Grip Strength	0.261	4.179***	-0.315	−4.929***	0.212	3.200**	−0.171	−2.442*	−0.265	−4.760***
1-back correct rate									−0.484	−7.444***
1-back response time									0.014	0.185
2-back correct rate									0.012	0.192
2-back response time									0.077	1.149
Age	−0.115	−1.813	−0.092	−1.425	0.057	0.843	−0.003	−0.044	0.147	2.698**
Years of education	0.386	6.697***	−0.333	−5.660***	0.325	5.306***	−0.165	−2.547*	0.009	0.159
R^2^	0.255		0.223		0.162		0.610		0.493	
F	27.556***		23.090***		15.522***		5.216**		32.872***	

Model 1: Grip strength predicts 1-back correct rate; Model 2: Grip strength predicts 1-back response time; Model 3: Grip strength predicts 2-back correct rate; Model 4: Grip strength predicts 2-back response time; Model 5: Grip strength, 1-back correct rate, 1-back response time, 2-back correct rate, and 2-back response time jointly predict ADL. * indicates *P* < 0.05; ** indicates *P* < 0.01; *** indicates *P* < 0.001.

**Figure 4:**
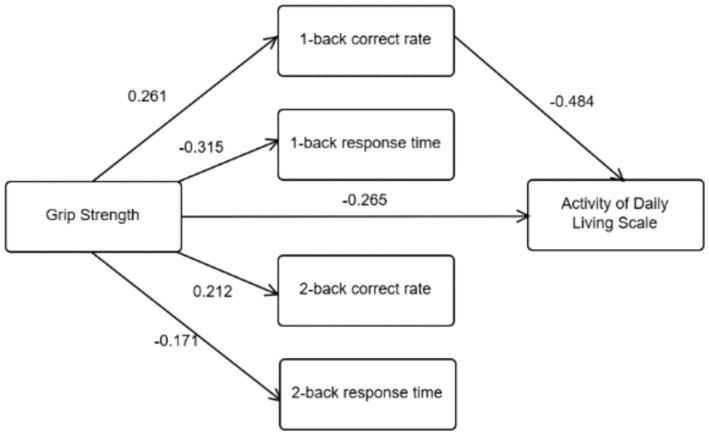
Structural relationship model of grip strength, working memory, and activities of daily living (ADL) (*N* = 245). Path values are standardized coefficients.

Controlling for age and years of education, further analysis using the bias-corrected Bootstrap method (Table 3) revealed that the total effect of grip strength on ADL was −0.175 (95% CI: −0.226 to −0.124), with the 95% confidence interval not crossing zero, indicating a significant effect. The direct effect of grip strength on ADL was −0.114 (95% CI: −0.161 to −0.067), also significant as the confidence interval did not cross zero. The mediating effect of 1-back correct rate was 0.054 (95% CI: −0.084 to −0.029), which again did not cross zero, indicating significance. However, the 95% confidence intervals for 1-back response time, 2-back correct rate, and 2-back response time all crossed zero, indicating non-significance. For details on the structural relationship model of grip strength, working memory, and daily living ability, refer to Figure 4. The path values represent the standardized coefficients between the pairs of variables shown in Table 2.

**Table 3:** Bootstrap analysis for significance testing of mediating effects (*N* = 245).

Variables			Bootstrap 95%CI	
	Effect Size	Boot SE	Lower limit	Upper limit
Total effect	−0.175	0.026	−0.226	−0.124
Direct effect	−0.114	0.024	−0.161	−0.067
1-back correct rate	−0.054	0.014	−0.084	−0.029
1-back response time	−0.002	0.016	−0.036	0.027
2-back correct rate	0.001	0.005	−0.009	0.012
2-back response time	−0.006	0.008	−0.026	0.004

#### 3.3.2 Structural relationship model of 30-s sit-up performance, working memory, and activities of daily living

Controlling for age and years of education, 30-s sit-up performance was taken as the independent variable, 1-back correct rate, 1-back response time, 2-back correct rate, and 2-back response time as the mediating variables, and activities of daily living as the dependent variable. As shown in Table 4, 30-s sit-up performance positively predicted 1-back correct rate (*B* = 0.523, *p* < 0.001) and 2-back correct rate (*B* = 0.386, *p* < 0.001), while negatively predicting 1-back response time (*B* = −0.421, *p* < 0.001) and 2-back response time (*B* = −0.214, *p* < 0.01). 30-s sit-up performance (*B* = −0.140, *p* < 0.05) and 1-back correct rate (*B* = −0.466, *p* < 0.001) negatively predicted ADL.

**Table 4:** Regression analysis of relationships among variables (*N* = 245).

Variables	Model 1		Model 2		Model 3		Model 4		Model 5	
	Beta	*t*	Beta	*t*	Beta	*t*	Beta	*t*	Beta	*t*
30-s sit-up	0.523	9.860***	−0.421	−7.131***	0.386	6.353***	−0.214	−3.169**	−0.140	−2.127*
1-back correct rate									−0.466	−6.476***
1-back response time									0.044	0.547
2-back correct rate									0.010	0.162
2-back response time									0.071	1.007
Age	−0.093	−1.795	−0.063	−1.093	0.065	1.091	0.017	0.261	0.233	4.464***
Years of education	0.247	4.661***	−0.239	−4.052***	0.225	3.704***	−0.119	−1.762	0.015	0.270
R^2^	0.431		0.294		0.252		0.076		0.455	
F	60.852***		33.445***		27.013***		6.629***		28.211***	

Model 1: 30-s sit-up performance predicts 1-back correct rate; Model 2: 30-s sit-up performance predicts 1-back response time; Model 3: 30-s sit-up performance predicts 2-back correct rate; Model 4: 30-s sit-up performance predicts 2-back response time; Model 5: 30-s sit-up performance, 1-back correct rate, 1-back response time, 2-back correct rate, and 2-back response time jointly predict ADL. * indicates *P* < 0.05; ** indicates *P* < 0.01; *** indicates *P* < 0.001.

**Figure 5:**
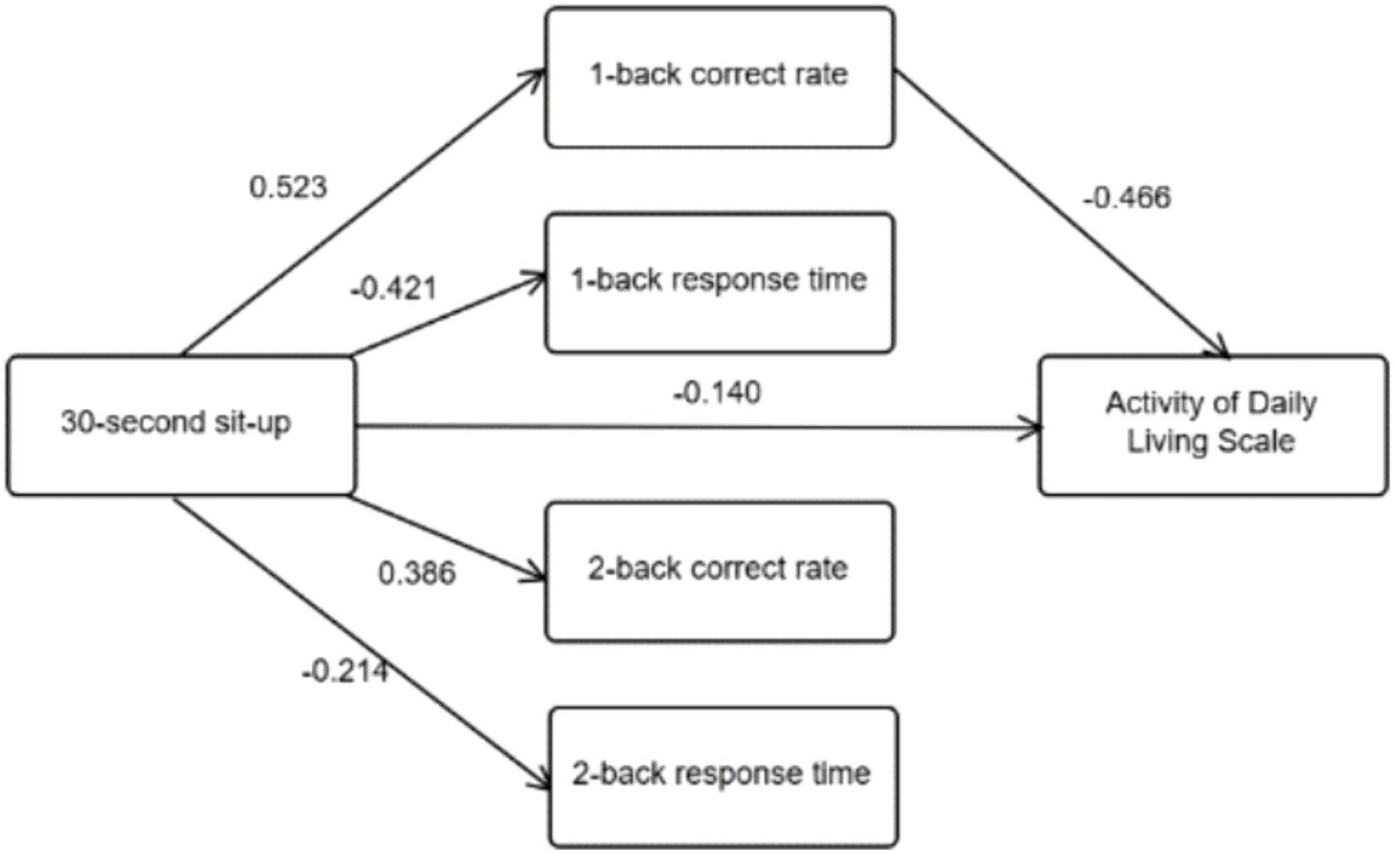
Structural relationship model of 30-s sit-up performance, working memory, and activities of daily living (ADL) (*N* = 245). Path values are standardized coefficients.

Controlling for age and years of education, further analysis using the bias-corrected Bootstrap method (Table 5) revealed that the total effect of the 30-s sit-to-stand on ADL was −0.280 (95% CI: −0.358 to −0.203), with the 95% confidence interval not crossing zero, indicating a significant effect. The direct effect of the 30-s sit-to-stand on ADL was −0.095 (95% CI: −0.183 to −0.007), also significant as the confidence interval did not cross zero. The mediating effect of 1-back correct rate was −0.166 (95% CI: −0.236 to −0.106), which again did not cross zero, indicating significance. However, the 95% confidence intervals for 1-back response time, 2-back correct rate, and 2-back response time all crossed zero, indicating non-significance. For details on the structural relationship model of the 30-s sit-to-stand, working memory, and daily living ability, refer to Figure 5. The path values represent the standardized coefficients between the pairs of variables shown in Table 4.

**Table 5:** Bootstrap analysis for significance testing of mediating effects (N = 245).

			Bootstrap 95%CI	
Variables	Effect Size	Boot SE	Lower limit	Upper limit
Total effect	−0.280	0.039	−0.358	−0.203
Direct effect	−0.095	0.045	−0.183	−0.007
1-back correct rate	−0.166	0.033	−0.236	−0.106
1-back response time	−0.013	0.037	−0.089	0.055
2-back correct rate	0.003	0.015	−0.027	0.031
2-back response time	−0.010	0.014	−0.042	0.011

## 4 Discussion

The results of this study indicate that greater muscle strength in older adults is associated with better daily living abilities, aligning with previous findings (Den Ouden et al., 2013; Gopinath et al., 2017; Jonkman et al., 2019). Muscle strength declines with age; research shows that muscle strength in middle-aged and older adults over 50 decreases by 12 to 14% every decade (De Carvalho et al., 2020). Higher muscle strength in this population is linked to a lower risk of impaired daily living abilities (Den Ouden et al., 2013; Gopinath et al., 2017; Jonkman et al., 2019), and changes in muscle strength May precede actual changes in daily living ability scores (Lau et al., 2023). This study found that grip strength has a direct effect on daily living abilities, whereas the 30-s sit-to-stand test does not. Grip strength is related to overall muscle strength (Bohannon, 2012) and is a predictor of overall mortality, cardiovascular diseases, respiratory diseases, and cancer (Celis-Morales et al., 2018; Leong et al., 2015). Grip strength affects the physical function of older adults (Norman et al., 2011); for every 5 kg decrease in grip strength, the risk of limitations in eating, walking, bathing, and toileting increases by 20, 14, 14, and 6%, respectively (Mcgrath et al., 2018). Lower limb muscle strength has a weaker effect on daily living abilities but May be a better predictor of fall risk (Simpkins and Yang, 2022; Jianming, 2019).

The study also found that greater muscle strength in older adults is associated with faster response times and higher correct rate in working memory tasks. Faster response times and higher correct rate in these tasks are linked to better daily living abilities. Higher muscle strength is associated with greater activation in specific prefrontal areas, leading to better working memory performance (Kilgour et al., 2014; Kobori et al., 2015). Grip strength is positively correlated with verbal and spatial abilities, processing speed, and memory in older adults (Firth et al., 2018a; Firth et al., 2018b). Among older adults experiencing cognitive decline, higher grip strength is related to better overall cognitive function and higher performance in declarative memory, working memory, and attention tasks (Filardi et al., 2022). Grip strength is influenced by the central nervous system (Rinne et al., 2018), and neural degeneration can lead to both cognitive decline and reduced grip strength (Kwon and Yoon, 2017). Lower limb muscle strength is closely related to attention and working memory task performance (Scherder et al., 2010); higher levels of lower limb muscle strength are associated with better cognitive and executive functions (Friedman et al., 2006; Anstey et al., 1997). In older women, insulin-like growth factor 1 (IGF-I) levels are positively correlated with lower limb muscle strength (Cappola et al., 2001). Higher IGF-I levels are associated with better overall cognitive function and performance in learning and memory tests (Calvo et al., 2013; Liqin et al., 2023), suggesting that IGF-I May mediate changes in muscle strength and cognitive function. Cognitive function is closely related to daily living abilities; even mild cognitive decline can negatively affect the ability to perform complex instrumental activities of daily living (Farias et al., 2003). Working memory is an important predictor of daily living abilities (Jacob et al., 2020); thus, better working memory is linked to better daily living abilities.

This study found that 1-back correct rate mediates the relationship between grip strength and daily living abilities, as well as the relationship between the 30-s sit-to-stand test and daily living abilities. Previous research has further demonstrated that the inhibitory component plays a major role in the differences between high and low working memory loads (Cong et al., 2017). The 1-back task reflects the recognition, maintenance, and updating of information in working memory, which involves a low memory load (Cong et al., 2017). In daily living abilities, basic activities of daily living (ADL) include fundamental personal self-care tasks such as eating, dressing, and grooming, while instrumental activities of daily living (IADL) include more complex tasks related to independent living, such as cooking, making phone calls, and managing finances. These tasks are closely related to low memory load working memory (Jian Wen-Jia et al., 2014). Conversely, high working memory loads that involve inhibition (e.g., 2-back tasks) are less prevalent in daily living abilities. In age-related decline, physical functions, represented by muscle strength, tend to decline first, subsequently affecting daily living abilities. There is a close relationship between muscle strength and working memory (Firth et al., 2018a); higher muscle strength is associated with better cognitive performance in older adults (Chen et al., 2015; Filardi et al., 2022). Declining muscle strength in older adults can impact their physical and social activities, which often involve interaction, discussion, and engagement with others, providing cognitive stimulation and opportunities for information processing. Reduced social engagement can mean less cognitive stimulation, possibly accelerating the decline in working memory. It is recommended that older adults engage in regular exercise tailored to their physical condition to increase muscle strength and improve both working memory and daily living abilities. Nursing homes and community centers should organize suitable physical and social activities, providing exercise guidance and facilities to help older adults participate in physical activities, enhance their physical and mental health, and promote social interaction, reducing feelings of loneliness and improving quality of life.

This study used a cross-sectional design; future longitudinal studies are needed to further explore the temporal relationships and effect sizes between muscle strength, working memory, and ADL. The sample size in this study was relatively small, limiting the generalizability of the results. Larger sample sizes are needed in future studies to verify these findings. Additionally, this study did not assess participants’ vitamin D levels or nutritional status, which May influence muscle strength and working memory. Future research should consider these factors.

## 5 Conclusion

There is a close relationship between muscle strength, working memory, and daily living abilities. Greater muscle strength is associated with better daily living abilities and working memory task performance. Low memory load working memory can mediate the relationship between muscle strength and daily living abilities. Appropriate physical exercise can enhance muscle strength in older adults, delay the decline in working memory, and help maintain or improve daily living abilities.

## Data Availability

The original contributions presented in the study are included in the article/supplementary material, further inquiries can be directed to the corresponding author.
